# The Actin Depolymerizing Factor (ADF)/Cofilin Signaling Pathway and DNA Damage Responses in Cancer

**DOI:** 10.3390/ijms16024095

**Published:** 2015-02-13

**Authors:** Chun-Yuan Chang, Jyh-Der Leu, Yi-Jang Lee

**Affiliations:** 1 Department of Biomedical Imaging and Radiological Sciences, National Yang-Ming University, Taipei 112, Taiwan; E-Mail: tracysirotan@hotmail.com; 2 Division of Radiation Oncology, Taipei City Hospital RenAi Branch, Taipei 106, Taiwan; E-Mail: dab03@tpech.gov.tw; 3 Biophotonics & Molecular Imaging Research Center (BMIRC), National Yang-Ming University, Taipei 112, Taiwan

**Keywords:** actin cytoskeleton, ADF/cofilin, radiosensitivity, DDR, radiotherapy

## Abstract

The actin depolymerizing factor (ADF)/cofilin protein family is essential for actin dynamics, cell division, chemotaxis and tumor metastasis. Cofilin-1 (CFL-1) is a primary non-muscle isoform of the ADF/cofilin protein family accelerating the actin filamental turnover* in vitro* and* in vivo*. In response to environmental stimulation, CFL-1 enters the nucleus to regulate the actin dynamics. Although the purpose of this cytoplasm-nucleus transition remains unclear, it is speculated that the interaction between CFL-1 and DNA may influence various biological responses, including DNA damage repair. In this review, we will discuss the possible involvement of CFL-1 in DNA damage responses (DDR) induced by ionizing radiation (IR), and the implications for cancer radiotherapy.

## 1. Introduction

According to the World Health Organization (WHO), cancer is a leading cause of morbidity and mortality globally. The occurrence of cancer in part correlates to the accumulation of DNA damage and loss or deterioration of normal genomic control [1]. A variety of strategies have been used to treat human cancers, but the efficacy of these approaches depends on the types of tumor cells [2,3,4]. An evidence-based analysis indicates that the utilization rate of radiotherapy is close to 52% compared to other therapies [5]. Although several novel biomedical techniques and new drugs have been developed to conquer cancers, radiotherapy remains an important primary or adjuvant method for cancer treatment.

In adherent cells, actin filaments are important for cell attachment and spreading on the substratum, and this process is required for cell proliferation. The significance of actin filaments is to convey the extracellular signals and form an appropriate shape for G1 phase progression [6,7,8,9,10,11]. Destabilization of the actin cytoskeleton using actin-targeting toxins (ATT) has been reported to cause reversible G1 phase arrest [12], although long-term treatment of these toxins would lead to apoptosis [13,14,15]. ATT was developed for chemotherapy, and considered in combination with radiation for cancer treatment [16,17,18]. However, the efficacy of different ATTs on the enhancement of radiosensitivity remains controversial. For instance, the G1 phase is sub-sensitive to ionizing radiation compared to G2/M phase arrest. Therefore, ATT-mediated accumulation of cells in the G1 phase may not be beneficial for increased radiosensitivity, except for the additional damage incurred by these cells. It is also possible that different types of ATT may trigger different molecular signaling pathways to respond differently to ionizing radiation. Clarification of these signaling pathways triggered by ATT may explain the conflict radiation responses induced by these compounds.

Regulation of the actin cytoskeleton is dependent on a variety of actin-associated proteins (AAPs). These AAPs accelerate the actin dynamics by polymerization and depolymerization of monomeric actin, as well as promotion of nucleotide exchange of ADP-bound actin. The members of the actin depolymerizing factor protein family are known to sever and/or depolymerize actin filaments* in vitro* and* in vivo* [19,20,21]. It is believed that these actions would replenish the actin pools and the quantities of actin filaments for actin cytoskeletal turnover. Accumulated reports have also shown that cofilin-1 (CFL-1), the primary non-muscle isoform of the ADF/cofilin protein family may also be involved in signaling transduction, nuclear entry of actin, and actin rod formation in the nucleus [22,23,24]. Our previous and recent studies also found that over-expression of CFL-1 could delay DNA repair after irradiation [25]. Interestingly, certain ATTs that differently affect CFL-1 activity also display distinct radiation response. In this review, we will discuss the role of CFL-1, actin cytoskeleton and other AAPs on DDR, and the implication of actin targeting for cancer radiotherapy.

## 2. The Actin Depolymerizing Factor (ADF)/Cofilin Signaling Pathways and Actin Dynamics

In the past three decades, studies of the ADF/cofilin family in various organisms have demonstrated that this molecule plays a crucial role in regulating actin dynamics, which affects locomotion, migration, and cell viability. The ADF protein was first identified from embryonic chick brain by Bamburg* et al.* [26], and related isoforms were subsequently found in a variety of organisms via functional assay and sequence similarity [27,28]. The first mammalian ADF protein was isolated from bovine brain extracts by Berl* et al.* [29], and subsequently found in the porcine brain extracts and kidney (also called destrin) [30,31]. In 1984, Maekawa* et al.* purified cofilin from porcine brain, and it is subsequently characterized to bind to actin subunits on F-actin in a 1:1 ratio, and it is named through the formed cofiliamentous structure with actin [30,32,33]. CFL-1 is classified as the non-muscle isoform of the ADF/cofilin protein family, and its full cDNA sequence was first cloned to deduce the amino acid sequence [34]. Although ADF and CFL-1 share 70% of sequence identity and they functionally overlap, CFL-1 is the major non-muscle isoform of ADF/cofilin in various cell types [35]. On the other hand, deletion of the *CFL-1* gene leads to lethality in mouse, yeast, fruit fly, and blastomere of Xenopus, suggesting that the functions of CFL-1 and ADF are not redundant [19,36,37]. Several important characteristics of CFL-1 will be discussed below.

### 2.1. Biophysics and Biochemistry of Cofilin-1 (CFL-1)

The fundamental function of CFL-1 is to accelerate the turnover of actin filaments by depolymerizing or severing the actin filaments. Interestingly, this action can enhance the actin polymerization for cell motility and other physiological behaviors. The depolymerization activity of CFL-1 can enhance actin dissociation from the pointed ends of actin filaments. The pointed ends of actin filaments contain ADP-bound actin subunits bound by CFL-1. Binding of CFL-1 opens the nucleotide-binding cleft of actin subunits on the filament, increases the average distance of adjacent actin subunits in the long-axis filaments and weakens the mutual interaction of actin subunits [38]. Therefore, the ADP-actin pool will be replenished by depolymerized actin that is subsequently converted to ATP-actin for the next round of polymerization. For the severing activity of CFL-1, the pointed end of actin filaments is occupied by CFL-1 to render a mechanical discontinuity of filaments [21]. The free barbed ends will increase by the severing activity of CFL-1, and they are the new sites for actin polymerization to enhance the actin dynamics [39]. Additionally, different concentrations of CFL-1 will also determine its action on nucleation or severing actin filaments [40]. Real-time fluorescent microscopic observation has shown that low CFL-1 concentration promotes severing of actin filaments, while high CFL-1 concentration decreases the extent of actin filamental severing [40,41]. When the CFL-1/actin ratio is high, actin-interacting protein 1 (Aip1) can bind to the CFL-1-actin filament and promote depolymerization of actin filaments to generate monomeric actins [42,43]. Additionally, CFL-1 binding to actin filaments is cooperative, but it actually does not affect the off-rate of actin filaments [44].

The function of CFL-1 on the turnover of actin filaments does not only rely only on the protein itself but also the environmental conditions. For example, growth factor-stimulated cell migration induces dramatic actin reorganization at the leading edge of cells, and this is associated with activation of CFL-1 via protein dephosphorylation and dissociation from phosphatidylinositol-4,5-bisphosphate (PtdIns(4,5)P2) [22]. Phosphorylation of the third serine residue (ser-3) on CFL-1 is believed to inactivate itself on actin depolymerization because the affinity between phosphorylated CFL-1 and actin is greatly reduced [45]. The ratio of phosphorylated CFL-1 to total CFL-1 is approximately 20%–50% in nontransformed mammalian cells [46,47]. CFL-1 can be phosphorylated by serine/threonine kinases including LIM kinase 1 (LIMK1), LIM kinase 2 (LIMK2) and testicular protein kinase 1/2 (TESK1/2) [48]. On the other hand, slingshot (SSH) phosphatase and chronophin can specifically dephosphorylate CFL-1 on ser-3. A recent report also proposes that tumor suppressor PTEN phosphates can directly dephosphorylate CFL-1 when prostaglandin E(2) (PGE(2)) is used to inhibit the phagocytosis of fungi, although it is not evident in mammalian cells [49]. In addition to protein phosphorylation, CFL-1 activity is also ablated by intracellular pH that is regulated by a sodium-proton exchanger on the cell membrane. CFL-1 will dissociate from cortactin or phosphatidylinositol 4,5-bisphosphate (PIP2) at higher pH, and the inhibitory effect on CFL-1 by these molecules will be removed [49]. Moreover, the subcellular localization of CFL-1, and regulation of actin dynamics by other AAPs such as profilin, tropomyosin and Aip1 will also directly or indirectly affect the activity of CFL-1 [39].

### 2.2. The Putative Role of CFL-1 in the Cell Nucleus

**Figure 1:**
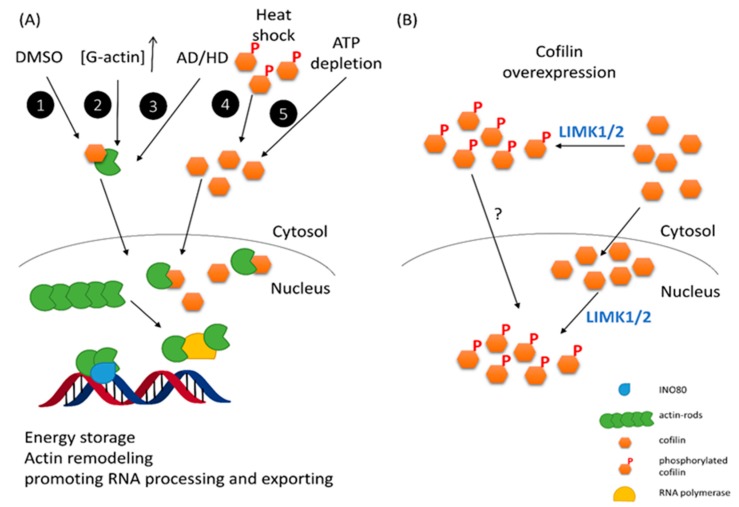
Nuclear entry of Cofilin-1 (CFL-1) via various stresses. (**A**) CFL-1 can bind G-actin and translocate to the nucleus to form actin rods by different exogenous and endogenous stresses. 1. 10% DMSO treatment; 2. Increase of G-actin concentration; 3. Neural degenerative diseases; 4. Heat shock stress; 5. ATP depletion. This action may avoid energy expenditure and promote chromatin remodeling via actin; (**B**) Enforced expression of CFL-1 can also occur in the nucleus. In this situation, phosphorylated CFL-1 is also detectable in nucleus, while the mechanism of nuclear entry of phospho-CFL-1 remains unclear. Because LIM kinase 1 (LIMK1) and LMK2 are responsible for CFL-1 phosphorylation in various cell types, the subcellular location of these kinases would determine the mechanisms of nuclear accumulation of phosphorylated CFL-1 and regulate actin dynamics in the cytosol and nucleus.

As one of the AAPs, CFL-1 is very different from others because it contains nuclear localization signals (NLS) in the protein sequence. This endows a particular ability of CFL-1 to bind and carry depolymerized actin to nucleus [50,51]. The NLS fragment of CFL-1 has been demonstrated to be functional when it is linked to nonnuclear proteins expressed in myotubes [51]. Recently, it was found that a conserved bipartite NLS, that is, two basic rich regions separated by seven amino acids (21-RKSSTPEEVKKRKK-34) played the functional role in mediating the nuclear localization of CFL-1 through the classic importin α/β interaction pathways [51,52]. When cells are faced with heat shock, ATP-depletion and dimethyl sulfoxide (DMSO) treatment, cytochalasin D or high cytosolic G-actin concentration, CFL-1 can translocate actin into nucleus and form actin rod-like structure [51,53,54,55,56]. Although the biological function of nuclear translocation of cofilin/actin is unclear, it may reduce the consumption of cellular energy because the ATP utilization in actin dynamics is tremendous. Sequestration of actins by cofilin in the nucleus would reduce actin dynamics in stressful condition and save cellular energy [57]. Although the dephosphorylated form of cofilin is essential for nuclear entry under these conditions, Nagaoka* et al.* have proposed that exogenous phosphorylated cofilin (S3D) is able to diffuse into the cell nucleus [58]. Our recent report also agrees that phosphorylatable, wild-type CFL-1 can be detected in cell nuclei by the tetracycline inducible gene over-expression system [25]. Of interest, a recent report showed that phosphorylated cofilin can transit from the cytoplasm to the nucleus in the laminar formation of the chicken optic tectum, indicating that phospho-cofilin plays a role in neural development biology [59]. On the other hand, it has been proposed that CFL-1 is phosphorylated in the nucleus by nuclear LIMK1 and LIMK2 [60,61]. Phosphorylated CFL-1 will be inactivated to depolymerize actin filaments, and nuclear actin rods are formed. A recent report has indicated that fluid shear stress can activate nuclear LIMK1/2 to phosphorylate cofilin in the nucleus and result in actin realignment for endothelial barrier integrity [62]. Moreover, the cell cycle regulator p57^kip2^ can bind to LIMK1, but not LIMK2, and translocate to the nucleus to reorganize actin fibers [63]. These mechanisms account for the importance of activated LIMK1/2 on the formation of phospho-CFL-1 in the cell nucleus. A summary of nuclear entry of cofilin via various stresses is represented in Figure 1. Taken together, the cytoplasm-nucleus transition of cofilin, either phosphorylated or unphosphorylated, is likely to play an essential role in various biological processes, and thus worthy of further investigation.

Although actin itself does not contain an NLS, it is well known that actin is important for chromatin remodeling by binding to INO80-associated chromatin modifying complexes [64]. Nuclear actin is also important for gene transcription by binding to RNA polymerase, promoting RNA processing and exporting mRNA to the cytoplasm [65]. It appears that ADF/cofilin may be an auxiliary molecule that transports actin to the cell nucleus for gene expression. The cytoplasmic ADF/cofilin may also influence gene expression via a nuclear actin independent pathway. For instance, ADF/cofilin-mediated actin turnover can promote the release of actin-bound nuclear transcription cofactors, such as the glucocorticoid receptor (GR) and serum response factor (SRF)-associated cofactor. Moreover, it has been reported that actin depolymerizing factor 9 (ADF9) of Arabidopsis controls the gene expression and muticellular development of plants [66]. We have performed a microarray analysis for mRNA and microRNA expression before and after induction of exogenous CFL-1 in human lung cancer cells, and we found many genes involved in cell cycle progression, amino acid metabolism, tumor suppression and even DNA damage response (DDR) are affected by over-expressed CFL-1 (unpublished data). Because CFL-1 does not contain a DNA binding domain, it is believed that the effects of CFL-1 on gene transcription would be indirect.

Nuclear entry of ADF/cofilin is usually induced by environmental stress. As mentioned, DMSO has long been known to trigger the nuclear entry of cofilin-actin complexes to form actin rods [54]. Additionally, heat-shock stress can induce nuclear translocation of CFL-1 through the NLS [24,52]. Formation of cofilin/actin rods in the nuclei and cytosol has also been detected in neural degenerative diseases including Huntington’s disease (HD) and Alzheimer’s disease (AD), respectively [67,68]. In neurodegenerative diseases, actin cytoskeletal regulation through cofilin is believed to be critical for the pathological etiology of these aging or stress related disorders [69]. Therefore, nuclear translocation of CFL-1 may play an important role in degenerative diseases. Whether this biological behavior of CFL-1 is to form specific actin-rod structures in the nucleus or to influence chromatin remodeling for gene expression is of interest for further study.

## 3. Genotoxicity and DNA Damage Response (DDR)

DNA damage can be induced by various environmental stresses, including ionizing radiation (IR), oxidative stress, ultraviolet (UV) light, and even polycyclic aromatic hydrocarbons in cigarettes. These genotoxicities cause different types of DNA damage followed by initiation of corresponding DNA repair systems. Additionally, DNA damage also triggers cell cycle checkpoints and initiates signaling pathways leading to apoptosis, senescence and even autophagy [70,71]. The elegant intracellular signaling networks activated by genotoxicity to determine the fate of insulted cells, such as cell cycle arrest, premature senescence, and apoptosis, is collectively termed the DNA damage responses (DDR). Because of the cell heterogeneity, the DDR in each single cell of one population may be different. For instance, the survival fraction is commonly used to determine the cell tolerance to genotoxicity, and this is one example of the evidence of cell heterogeneity [72,73]. Ionizing radiation induced cell death is usually caused by unrepairable DNA damage. A functional DNA repair system would increase viability of irradiated cells, although it may leave errors in the DNA sequence and lead to mutation. Here we primarily focus on the ionizing radiation induced DDR and the potent effect on clinical radiotherapy [74].

### 3.1. Types of Ionizing Radiation on Cell Survival and DDR

**Figure 2:**
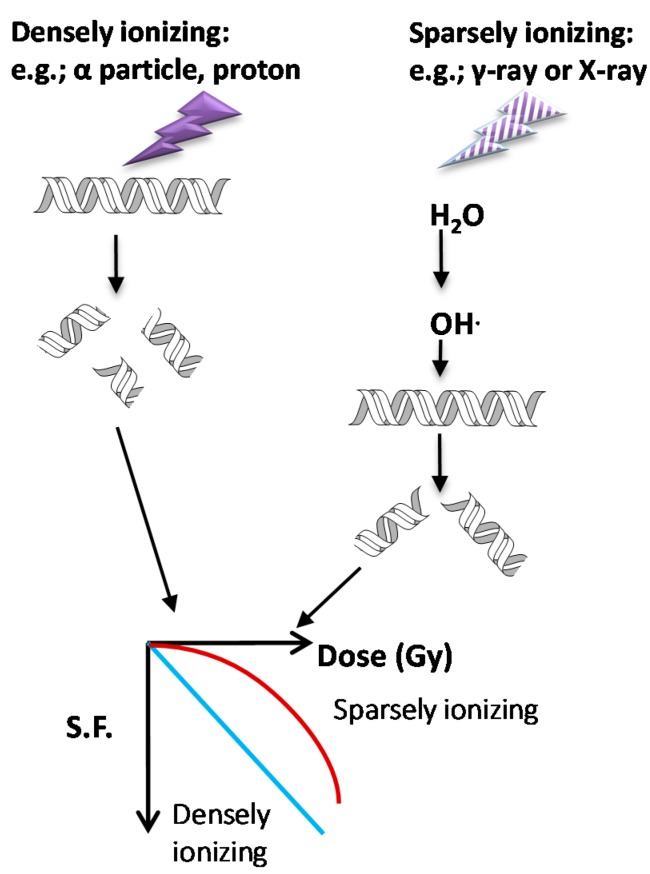
Effects of different radiation types on DNA damage and cell survivals. Particulate radiations by α-particles or protons belong to densely ionizing radiation type because they can deposit most energy on their tracks. On the other hand, X-rays and γ-rays possess high penetration ability but leave little energy on the traveling track, so that they are sparsely ionizing radiation. Moreover, sparsely ionizing radiation mainly ionizes water to produce free radicals, which are highly electrophilic and prone to capture electrons from biological components. When DNA locates on the track of these two different radiation types, it displays different levels of damage. Densely ionizing radiation causes more double strand breaks than sparsely ionizing radiation, so that DNA repair will be accordingly less efficient. Under the same dosage, the survival fractions of particulate radiation irradiated cells will be lower than that of X-ray or γ-ray irradiated cells.

Ionizing radiation is generated by either high-energy photons or particles that can eject electrons or break the nucleus of the atom. Thus, the incident energy is to “ionize” charged particles that are originally bound in the atom, including secondary electrons and recoiled protons. X-ray and γ-rays belong to electromagnetic radiation with photon properties, while α particles and β particles belong to particulate radiation that can carry either a positive or negative charge. X-rays and γ-rays are regarded sparse types of radiation because they can easily penetrate through the object and only deposit little energy in it. On the other hand, α particles and protons are dense types of radiation that have difficulty to penetrate the object but can deposit large amounts of energy in it. X-rays and γ-rays can only generate ionized fast electron, which has the mass 1/2000 and 1/8000 of proton and α particle, respectively. Therefore, it is easy to understand that particulate radiation usually causes higher biological damage than electromagnetic radiation. X-rays and γ-rays generate fast electrons that can interact with water to generate free radicals that is the source of oxidative stress to damaged DNA. This is a so-called indirect effect. On the contrary, protons and α particles can directly deposit their energy on DNA even though they can also generate oxidative stress, and this is called a direct effect. Both effects are sufficient to break the covalent bonds of DNA and result in DNA double strand breaks (DSB), but the levels are more severe by protons or α particles than by X-rays and γ-rays under the same absorbed dosage. The levels of DSB are reflected through measuring the survival fractions which represent the tolerance of cells to different types of radiation. The lower survival fraction caused by particulate radiation is related to complex DNA damage that is difficult to be repaired. The survival curve is drawn according to the survival fractions corresponding to increased radiation dose. In a semi-log plot of survival curve, α particles or protons usually exhibit a linear curve that stands for exponential killing, but X-rays and γ-rays will give a “shoulder” at lower doses before the appearance of the exponential killing curve. The “shoulder” region of the survival curve indicates that at low dose radiation, cells would repair effectively after irradiation by X-rays and γ-rays. However, there is no, or a very narrow, shoulder of survival curve observed using α particles or proton irradiation under the same dose. A scheme represents the effects of radiation types on DDR and survival curves are shown in Figure 2.

### 3.2. DNA Repair Mechanisms Following Ionizing Radiation (IR)

DNA double-strand breaks (DSB) are the major type of DNA damage after exposure to ionizing radiation. DSB damage needs to be repaired to maintain genomic stability and survival. In mammalian cells, the DNA repair mechanisms include nonhomologous end joining (NHEJ) and homologous recombination repair (HRR), in which different DNA DSB repair proteins are involved. The key molecules for NHEJ include DNA end binding proteins Ku70/Ku80 heterodimer, which in turn binds and enhances DNA-dependent protein kinase (DNA-PKcs) activity [75,76,77,78]. Artemis protein, an exonuclease involved in V(D)J recombination for immunological responses, can gain endonuclease activity after joining a complex with DNA-PKcs to process the broken end of DNA after IR. Finally, the DNA ends are filled in and connected through DNA polymerase λ/μ and PNK/XRCC4/DNA ligase IV/XLF complex, respectively. This error-prone repair system mainly occurs in G0, G1 and early S phase because no replicative template is required for repairing DSB [79]. For HRR, *E. coli* recombinase RecA-homologous protein Rad51 and its interacting partner Rad52 are important for single-strand DNA binding, strand exchange, and annealing using the sister chromatid as the repair template [80,81,82,83]. The strand exchange or invasion is assisted by BRCA1/2 tumor suppressor proteins, and Rad54 is a helicase that can hydrolyze ATP to unwind the double-strand DNA in template. After completion of repair, the tangled DNA structures, so called Holiday junctions, are resolved by MMS4/MUS81 hetermodimer with endonuclease activity. Overall, this is an error-free repair system that is believed to be involved in late S and the G2 phase of the cell cycle. Deficiency of Ku70 or Ku80, or inhibition of Rad51 levels can lead to enhancement of radiosensitivity [78,84,85,86]. Moreover, over-expression of Rad52 confers resistance to ionizing radiation in mammalian cells [87]. Therefore, cell radiosensitivity is strongly associated with DNA repair capacity [86,88,89,90]. Increased cell radiosensitivity by repressing DNA repair capacity is one of the important strategies for design of radiosensitizers.

### 3.3. The Role of the Mre11-Rad50-NBS1 (MRN) Complex in Sensing IR-Induced DNA Damage

Although NHEJ and HRR utilize different molecules to directly execute the re-joining of broken DNA ends, they have the DSB sensor in common. The MRN complex comprised by Mre11 (meiotic recombination 11), Rad50 and NBS1 (Nijmegen breakage syndrome 1) is reported to recognize the sites of DSB to trigger both NHEJ and HRR [91]. The MRN complex is also known to maintain the integrity of telomeres of chromosomes in eucaryotic cells [92]. Mre11 can specifically bind to DSB termini and perform single-strand DNA (ssDNA) endonuclease and 3' to 5' double-strand DNA (dsDNA) exonuclease activities [93]. Rad50 can bind to Mre11 and form a core tetramer complex* in vivo* [94]. Rad50 can unwind dsDNA in DSB ends by hydrolyzing ATP [95]. NBS1 does not contain enzymatic activity, but is important for transport oft he MRN complex into the nucleus [96]. It also mediates the DNA repair response by promoting protein-protein interaction at DSB sites. Importantly, both NBS1 and Rad50 can be phosphorylated by Ataxia telangiectasia mutated (ATM) serine/theronine kinase for intra-S phase checkpoint and repair in response to IR [97,98,99]. However, ATM mediated phosphorylation of the MRN complex does not guide the MRN complex to DSB termini. On the contrary, this phosphorylation is required to recruit ATM to DSB termini for further activity. Binding of the MRN complex to DSB sites is important for both classical NHEJ (C-NHEJ) and alternative NHEJ (A-NHEJ, without XRCC4, Ku, and DNA-PKcs activity) because knockdown of Mre11 can impair both pathways [100]. Furthermore, the MRN complex is also essential for processing DSB termini for Rad51/Rad52 binding in HRR.

### 3.4. Ignition of DDR by Ataxia Telangiectasia Mutated (ATM) Kinase

ATM is a member of phosphatidylinosital 3-kinase related kinase (PIKKs) superfamily that also includes ATR (ATM- and Rad3-related), DNA-PKcs, mTOR (target of rapamycin), SMG1 (suppressor with morphological effect on genitalia), and TRRAP (transformation/transcription domain-associated protein). Recruitment of ATM to DSB is dependent on MRN complex, although this DSB sensor may be not required for C-NHEJ under low dose or low-LET irradiation [101,102]. ATM has been regarded a transducer following the MRN complex that functions as a sensor of DSB [103,104]. In addition to the DSB site, genotoxicity-induced chromatin relaxation around DSB is also important for activation of ATM. The chromatin surrounding DSB will be modified by PARP-1 (poly (ADP-ribose) polymerase 1) that introduces poly(ADP-ribose) chains (PAR chains) to histone H1 and histone H2B [105,106]. Several chromatin remodeling molecules including PcG (polycomb group), NuRD (nucleosome remodeling deacetylase), ALC1 (amplified in liver cancer 1), and HP1/-KAP-1 (H3-trimethyl K9 binding protein 1-KRAB associated protein 1) will interact with PARylated chromatin encompassing DSB for further processes [107,108,109,110]. These chromatin relaxation procedures mainly promote posttranslational modification of DSB and histones through ubiquitination, stabilization of chromatin structure, and unpacking of heterochromatic DSB [109,110,111,112]. ATM is also PARylated and activated by PARP1 after IR-induced DSB. Inhibition of PARP1 reduces ATM activity and DNA repair capacity, and it has been considered for design of cancer radiotherapeutic strategy [113,114].

Over 700 protein targets have been identified as phosphorylated by ATM and/or ATR after genotoxic treatment according to large-scale proteomic screening and analysis [115,116,117]. ATM itself is the substrate of this protein, and serine 1981, 367 and 1893 (ser1981, ser367, ser1893) are the autophosphorylation residues after IR-induced DSB [118]. As mentioned, the MRN complex is important for recruitment of ATM to the DSB sites. The lack of a MRN complex leads to the reduction of ser1981 phosphorylation and activity of ATM. The phosphorylation kinetics of ser1893 is slower than that of ser1981 of ATM. ATM phosphorylation on ser1893, unlike ser1981, fully depends on the MRN complex. This may explain the different phosphorylation kinetics between these two serines on ATM [118]. Activated ATM at DSB termini will further phosphorylate H2AX (γ-H2AX), NBS1, BRCA1 (breast cancer type 1 susceptibility protein), MDC1 (mediator of DNA damage checkpoint 1) and SMC1 (structural maintenance of chromosome 1) to promote DNA repair [119,120]. It is noteworthy that MDC1 can bind both γ-H2AX and NBS1 to cooperatively increase ATM activity for formation of γ-H2AX over megabases surrounding DSB sites [121,122,123]. 53BP1 is a scaffold for DSB responsive factors, and can be phosphorylated by ATM to increase the substrate-specificity of ATM [124,125,126]. When ATM is not bound to DSB sites, it can phosphorylate p53 and Chk1/2 to influence cell cycle checkpoint and apoptosis. Therefore, ATM is a critical center for DDR in response to genotoxicity.

**Figure 3:**
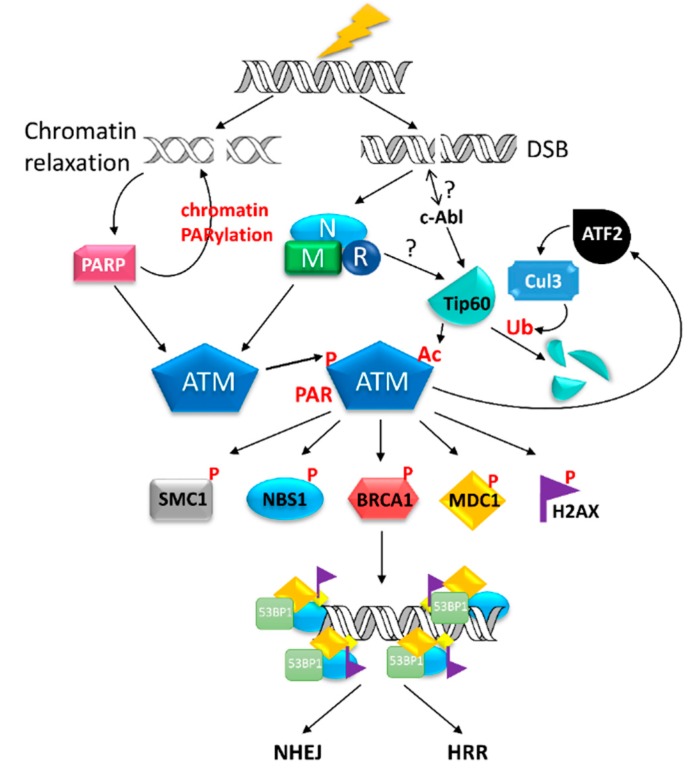
DNA damage-induced molecular responses for DNA repair. In response to ionizing radiation, DNA double strand breaks (DSB) and chromatin relaxation surrounding DSB sites will occur. The MRN complex can sense and bind to DSB sites and quickly recruit ATM kinase for autophosphorylation and paraphosphorylation to various molecules, including NBS1 and Rad50 in the MRN complex. In addition, chromatin relaxation will activate PARP1 to execute PARylation on chromatin associated molecules and ATM kinase. Activated ATM needs to be maintained by acetylation via HAT Tip60, which is negatively regulated by the ATF2-Cul3 protein degradation pathway. Although c-Abl kinase can activate Tip60 at the DSB site, how DSB interacts with c-Abl is unclear. Additionally, whether MRN complex will regulate Tip60 to sustain ATM activity is unknown. ATM also regulates ATF2 activity, and it is likely to be an autoregulatory mechanism of ATM activity. Activated ATM can phosphorylate SMC1, NBS1, BRCA1, MDC1, 53BP1, and H2AX to localize DSB sites for further DSB repair mechanisms, including HRR and NHEJ.

Recruitment and autophosphorylation of ATM at the DSB site after IR damage requires further activation to maintain the ATM activity for substrate phosphorylation. That is, ATM needs to be further modified by other mechanisms in addition to phosphorylation. The histone acetyltransferase (HAT) Tip60 is recruited and bound to ATM by the MRN complex in response to DSB [127,128]. Tip60 can acetylate ATM at lys3016 and is required for ATM-mediated phosphorylation on H2AX, p53 and Chk2 [118]. Tip60 is further activated by c-Abl tyrosine kinase at the DSB site [128,129]. Additionally, Tip60 is strictly controlled by ATF2 (Activating Transcription Factor 2), a bifunctional transcription factor that can promote Tip60 ubiquitination via E3 ubiquitin ligase Cul3 and proteasomal degradation to influence global DSB repair [130]. Knockdown of ATF2 can stabilize Tip60 and lead to activation of ATM and increase cell survival after IR damage [131]. On the other hand, ATM can phosphorylate ATF2 on ser490/ser498 and confer the transcription-independent activity of ATF2 that can bind to γ-H2AX and MRN complex for S phase checkpoint [130]. Under this condition, inhibition of ATF2 leads to loss of S phase checkpoint and reduction of DNA repair capacity as well as ATM activity. Interestingly, Ser490A/ser498A phospho-mutant ATF2 cannot influence Tip60 stability after over-expression. This implies that ATM will negatively regulate its own activity by phosphorylating ATF2 to ablate Tip60, which may occur after DNA repair is completed. The interplay between c-Abl-Tip60 and ATF2 would be essential to regulate ATM activity after they are recruited to DSB following IR [129,132,133]. A scheme of DNA damage-induced molecular responses for DSB repair is shown in Figure 3.

## 4. Actin Dynamics, ADF/cofilin and DDR

Although genotoxicity is meant to disrupt DNA structures, accumulated literature suggest that the genotoxicity induced DDR is not only associated with DNA breaking itself but also the reorganization of the actin cytoskeleton [134,135,136]. The expression and activity of AAPs are of interest to be investigated because their activity and/or subcellular distribution are affected by genotoxicity. These effects largely influence DDR, including apoptosis and cell cycle progression by interacting with other DNA damage induced molecules, such as the p53 tumor suppressor protein. Notably, destabilization of the actin cytoskeleton using ATTs or deliberate manipulation of AAP expression can also modulate genotoxicity-induced DDR. In this section, how actin dynamics and AAPs play a role in response to DNA damage will be discussed.

### 4.1. Actin Response Following DNA Damage

DNA damage can induce actin reorganization that influences apoptosis and cell cycle arrest subsequently. For instance, use of synchrotron radiation X-ray scattering, actin in solutions of purified calf spleen actin will polymerize without a lag phase [137]. Induction of transient actin polymerization and later actin depolymerization in HL-60 cells is important for ultraviolet (UV) irradiation or etoposide-induced apoptosis [138]. Of interest, cytolethal distending toxins (CDTs), a protein toxin generated by Gram-negative bacteria, are able to induce DNA damage and promote the formation of actin stress fibers [139]. However, the formed stress fibers are due to RhoA GTPase activation, which is mediated by a guanine nucleotide exchange factor (GEF), so-called neuroepithelioma transforming gene 1 (Net1), that also promotes the p38 mitogen-activated protein kinase (MAPK) pathway to extend cell survival [139]. This effect partially suggests that chronic exposure of genotoxicity will lead to genomic instability and mutation. Actin polymerization induced by DNA damage is also associated with p53. It has been reported that LIMK2b, a potent tumor suppressor, is induced by p53 to modulate actin dynamics for executing G2/M arrest through cofilin phosphorylation after DNA damage [140]. On the other hand, p53 mediated transaction of RhoC-LIMK2 leads to inactivation of cofilin, which reduces the actin depolymerization and leads to an increase of actin stress fibers. Under this condition, enhanced actin cytoskeletal formation promotes cell survival, and the efficacy of DNA damage-related therapy will be compromised [136]. The combination of a LIMK2 inhibitor and genotoxic therapy has potential for cancer treatment because it is impossible to inhibit p53 to prevent radio-chemoresistance of cancer cells. It appears that different splicing variants of LIMK2 transactivated by p53 would lead to distinct cell fates, and it requires careful investigation to clarify the effects of actin cytoskeletal remodeling mediated by the p53-LIMK2 pathway.

**Figure 4:**
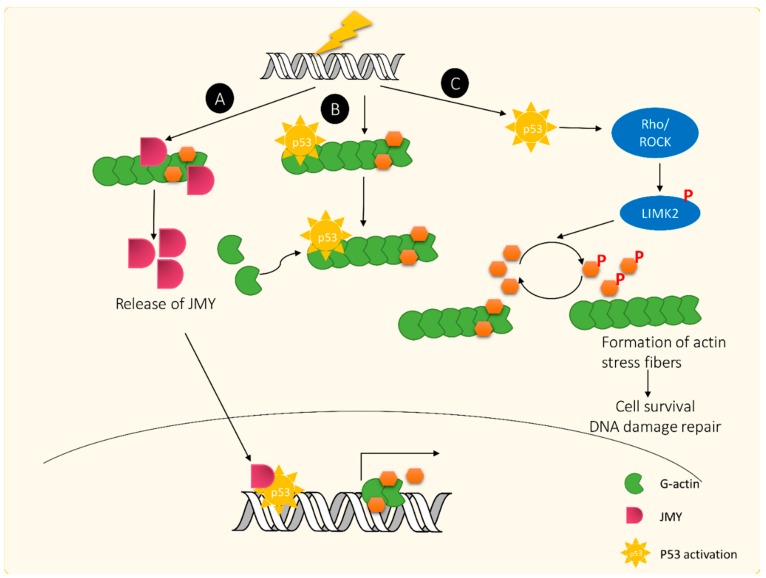
Regulatory mechanisms of actin dynamics in response to DNA damage. (**A**) Radiation-induced actin polymerization triggers the release of JMY from G-actin. JMY then enters the nucleus and binds to p53 for p53-dependent apoptosis; (**B**) When DNA damage occurs, polymerized actins may allow p53 binding to actin filaments and retain p53 in the cytoplasm. DNA repair would dominate p53-mediated apoptosis under this condition; and (**C**) DNA damage-mediated actin polymerization would be caused by p53 induction of the Rho/RCOK signaling pathway, followed by activation of LIMK2 to phosphorylate and inactivate cofilin. The actin depolymerization rate will be reduced to maintain polymerized actins.

In another aspect, DNA damage-induced actin polymerization also negatively regulates p53 function by localizing p53 in the cytoplasm [135]. This effect may delay p53 mediated apoptotic processes and allow DNA repair to be processed before apoptosis. Indeed, it has been reported that polymerized actin is required for DSB repair [141]. Binding of p53 to actin filaments is calcium dependent, and the interaction between these two entities is enhanced by DNA damage [142]. On the other hand, p53 mediated gene transcription is also dependent on actin polymerization. A G-actin binding protein, so-called junction-mediated and regulatory protein (JMY) is released from polymerized actin following DNA damage. The free JMY containing a bipartite NLS enters the nucleus and interacts with p53 to enhance its transcriptional activity for DDR [134,143]. The potent actin cytoskeletal responses to DDR are summarized in Figure 4.

### 4.2. DDR Following Destabilization of the Actin Cytoskeleton

Since DNA damage promotes actin polymerization for subsequent biological responses, it is speculated that destabilization of the actin cytoskeleton will influence DDR [141]. Because the actin cytoskeleton is a dynamic structure, forced polymerization or depolymerization of actin will lead to destabilization of the actin cytoskeleton. Additionally, insufficient energy flux will impair nucleotide exchange of monomeric actin, a rate-limiting step of actin dynamics, to destabilize the actin cytoskeleton. Actin targeting toxins (ATTs) are extracted from fungi or sea sponges, and the biochemical functions of actin filaments are different among these ATTs (Table 1). Cytochalasin D (CD) has been reported to disrupt actin microfilaments and activate p53, which causes G1-to-S phase arrest and apoptosis [15]. It is unclear whether CD-induced p53 activity is the primary or secondary effect of microfilamental disruption since p53 up-regulation is largely dependent on DNA damage. On the other hand, disruption of actin filaments by cytochalasin B (CB), CD, and latrunculin B (LB) can induce cell cycle regulator p21^CIP1/WAF^ in p53-null cells [144]. Interestingly, actin inhibitors-induced p21^CIP1/WAF^ can be detected before p53 activation in p53-wild-type cells, suggesting that disruption of actin filaments by actin inhibitors would affect cell growth without DNA damage. Disruption of actin filaments using Rho inhibitors or actin inhibitors also increases p21^CIP1/WAF^ protein stability, which has been reported to be caused by activation of the c-Jun *N*-terminal kinase (JNK) stress activated protein kinase (SAPK) pathway [145]. Additionally, disruption of the actin cytoskeleton can activate the JNK pathway via the mammalian Ste20-like (MST) kinase, which can stabilize the p21^CIP1/WAF^ via a JNK dependent phosphorylation on Thr57 [146]. MST kinase actually is required for stabilization of p21^CIP1/WAF^ after disruption of the actin cytoskeleton. Therefore, it is of interest to further investigate whether disruption of the actin cytoskeleton increased p21^CIP1/WAF^ stability via the MST-JNK pathway is also independent of p53.

**Table 1:** The sources and functions of various actin inhibitors.

Actin Inhibitors	Source/Host	Function
Cytochalasin B (CB)	*Helminthosporium dematioideum*/fungi	Blocking monomer add-on at the fast-growing end of actin filament
Cytochalasin D (CD)	*Zygosporium mansonii*/fungi	Blocking monomer add-on at the fast-growing end of actin filament, 10-fold more potent than CB
Latrunculin A	*Latrunculia magnifica*/Red Sea Sponge	Formation of a 1:1 complex with monomeric G-actin (*K*_d_ = 200 nM)
Misakinolid A (Bistheonellide A)	*Theonella* sp./marine sponge	Inhibits actin polymerization by forming a 1:2 complex with G-actin
Mycalolide B	*Mycale* sp./marine sponge	Severs F-actin and forms a 1:1 complex with G-actin to sequester it; it also suppresses actin-activated myosin Mg^2+^-ATPase activity
Swinholide A	*Theonella swinhoei*/marine sponge	Sequestering actin dimers with a binding stoichiometry of 1:1, and rapidly severing F-actin
Jasplakinolide	*Jaspis johnstoni*/marine sponge	A potent inducer of actin polymerization and stabilization* in vitro*, cell-permeable
Phalloidin	*Amanita phalloides*/fungi	A potent and specific F-actin binding agent; Inhibitor of F- to G-actin conversion, cell non-permeable

Morphological change is one of the most important characteristics of apoptosis induced by DNA damage. Disruption of actin microfilaments or microtubules has been reported to accelerate actinomycin D (AD)-induced DNA damage and apoptosis, but forced stabilization of these cytoskeletons has no such effects [147]. Pretreatment of cells with latrunculin or CD followed by ionizing radiation shows that the NHEJ-related proteins Ku70/Ku80 are reduced to bind to the DNA ends caused by IR [141]. Jasplakinolide (JP), an actin stabilizing reagent, has been reported to exhibit an additive effect to radiation-treated prostate cancer cell lines [148]. We have used the colony formation assay, a gold standard of radiobiological research to investigate the effects of CB and latrunculin A (LA) on radiosensitivity. The data revealed that LA but not CB could enhance radiosensitivity [25]. Cellular γ-H2AX is induced by radiation combining CB but not LA, suggesting that LA-mediated destabilization of the actin cytoskeleton would interfere with H2AX phosphorylation induced by IR [25]. Additionally, LA and CD have been reported to increase the level of ser-3 phosphorylated cofilin [149], whereas we found that CB did not show this ability. Whether actin inhibitors influence the expression of certain AAPs to influence DDR is of interest to further investigate.

### 4.3. AAPs and DDR

Actin dynamics are ablated by a variety of AAPs. Although AAPs regulate actin organization in the cytoplasm, accumulated literature has demonstrated that several AAPs can shuttle between the cytoplasm and the nucleus to promote the formation of nuclear actin polymers and/or affect gene transcription. Moreover, they are important for the DNA repair system. Human actin-related proteins 5 (hArp5) predominantly localizes in the nucleus and associates with chromatin remodeling molecules INO80 to promote DNA repair through accumulation of γ-H2AX [150,151,152]. JMY also exists in both the cytoplasm and nucleus, while its level in the nucleus will increase following DNA damage [153]. Increase of JMY accumulation in the nucleus is actin dependent and required for p53 activation [143]. Filament A (FLNA), alternatively named actin-binding protein 280 (ABP-280), cross-links cortical actin filaments into a firm 3D structure. Interestingly, FLNA also binds DNA repair proteins BRCA1/2 for HRR [154,155,156]. Inhibition of FLNA can increase the radiosensitivity and chemosensitivity to IR and cisplatin, respectively [80]. The G-actin sequestering protein thymosine β4 (Tβ4) can bind to Ku80 to regulate the expression of plasminogen activator inhibitor type 1 [157], though it is unclear whether this interaction is essential for DNA repair. Although the actin nucleator, neural Wiskott–Aldrich syndrome protein (N-WASP), has not been reported to be related to DDR, its activator, so-called non-catalytic region of tyrosine kinase adaptor protein 1 (NCK1) can translocate to the nucleus to activate p53 [158]. Nuclear NCK1 also associates with the suppressor of cytokine signaling 7 (SOCS7) and G-actin to influence actin cytoskeletal reorganization in response to DNA damage [158].

### 4.4. ADF/Cofilin and DDR

**Figure 5:**
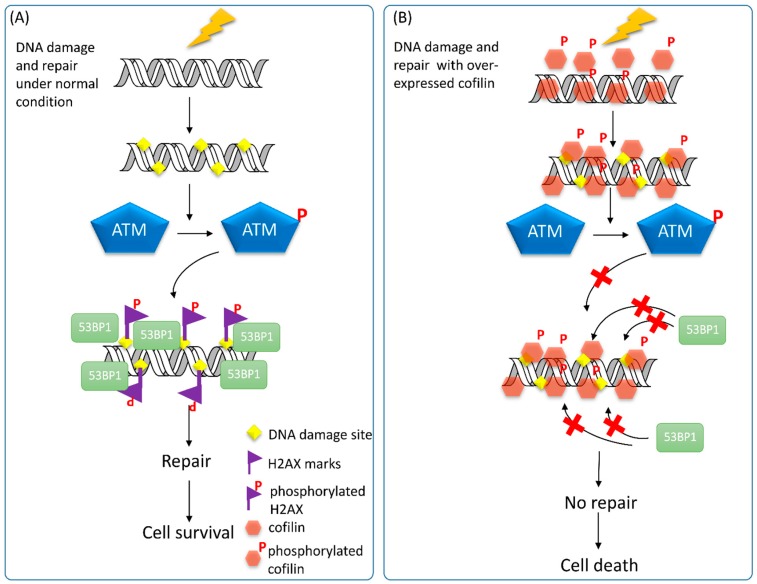
The putative effects of over-expressed CFL-1 on DNA repair. (**A**) Under normal condition, IR-induced DNA damage will trigger the activation of the ATM kinase, which phosphorylates H2AX encompassing DNA damage sites. 53BP1 will recognize γ-H2AX and recruit additional DNA repair molecules to fix the damaged DNA and lead to cell survival; and (**B**) Over-expression of wild type CFL-1 (phosphorylatable) may enter and locate around DNA. After IR, the ATM kinase remains to be activated, but cannot phosphorylate H2AX. Recruitment of 53BP1 to the γ-H2AX sites fails, so that the DNA repair capacity is impaired and results in cell death.

The effects of CFL-1 on DNA repair and radiosensitivity have been previously established. Over-expression of wild-type, S3A and S3D mutant CFL-1 all lead to enhanced radiosensitivity, but ρ-activated kinase (ROCK) inhibitor Y27632 does not increase radiosensitivity of treated cells [25]. Interestingly, γ-H2AX could not be detected in CFL-1 over-expressing cells after irradiation, whereas the ATM activity was not affected under this condition. Therefore, we hypothesize that over-expressed CFL-1 may enter the nucleus and hamper recognition of H2AX by ATM kinase, and this phenomenon would lead to failure of DNA repair initiation (Figure 5). As mentioned, the actin inhibitor LA but not CB can induce both cofilin phosphorylation and enhanced radiosensitivity that is associated with impaired formation of γ-H2AX [25]. This observation implies that the phospho-state of CFL-1 is involved in regulating the cellular radiosensivity. Because the phosphorylation of cofiln-1 is directly controlled by the Rho-LIMK pathway and SSHL1 phosphatase, it will be better to elucidate the role of phosphorylated CFL-1 on DNA repair and radiosensitivity by manipulating the expression of the LIMK and SSHL1 gene. On the other hand, Yan* et al.* reported that in radioresistant astrocytoma, CFL-1 is one of the proteins up-regulated in patients [159]. However, we have previously found that the CFL-1 level of human A549 lung adenocarcinoma is higher than that of H1299 cells [160], which exhibit stronger radioresistance than A549 cells [161]. Our recent data using* in vivo* isolated breast cancer stem cells also exhibit lower CFL-1 levels and higher radioresistance than parental breast cancer cells (manuscript in preparation). Therefore, the association of CFL-1 and radiation-induced DDR may be cell- or tissue-dependent. A global survey of CFL-1 expression on different types of cancer sources would be essential to design the therapeutic strategy to utilize the cofilin signaling pathway for cancer radiotherapy.

## 5. Conclusions

The cross-talk between the cytoplasm and nucleus is increasingly important in upcoming bio-research phases. The actin cytoskeleton and associated proteins, originally support cellular mechanical functions in the cytoplasm, and have been regarded as critical messengers and cofactors to modulate the extracellular and intracellular signaling to the nucleus, a sanctuary of eukaryotic cells to orchestrate cell responses. Although DDR is believed to be associated with the dynamic architecture of the cytoskeleton, the role of different AAPs on regulation of DDR following genotoxicity remain largely unknown. As a primary regulator of actin dynamics and an essential gene to ablate cell viability, CFL-1-mediated DDR is of interest to be investigated. At a minimum, several conflicting results from different research groups need to be clarified in the future. It is expected that these studies would define the role of actin-targeting agents for radiotherapy.
